# Risks of Cervical Cancer Recurrence After Fertility-Sparing Surgery and the Role of Human Papillomavirus Infection Types

**DOI:** 10.3390/jcm13216318

**Published:** 2024-10-22

**Authors:** Gulzhanat Aimagambetova, Gauri Bapayeva, Talshyn Ukybassova, Nazira Kamzayeva, Gulnara Sakhipova, Nasrulla Shanazarov, Milan Terzic

**Affiliations:** 1 Department of Surgery, School of Medicine, Nazarbayev University, Astana 010000, Kazakhstan; 2 Clinical Academic Department of Women’s Health, CF “University Medical Center”, Astana 010000, Kazakhstantalshynu@yandex.ru (T.U.);; 3 Department General Practitioners, West Kazakhstan Medical University, Aktobe 030000, Kazakhstan; 4 Center for Photodynamic Therapy, Medical Center Hospital of The President’s Affairs Administration of The Republic of Kazakhstan, Astana 010000, Kazakhstan

**Keywords:** cervical cancer, early-stage cervical cancer, HPV type, HR-HPV, fertility-sparing surgery, recurrence

## Abstract

Cervical cancer is a largely preventable malignancy of the uterine cervix. The tendencies in cervical cancer morbidity and mortality have remained similar for the past decade, albeit with increasing frequency in low- and middle-income countries (LMICs). Moreover, in the majority of LMICs, cervical cancer is the second most prevalent cancer and the second most common cause of cancer-related death among reproductive-age women. High-risk human papillomavirus (HR-HPV) infections have been proven to be associated with up to 95% of cervical cancer cases, with HPV-16 and HPV-18 types being responsible for approximately 70% of all cervical cancers, with the other high-risk HPV types accounting for up to a further 25%. More recently, the latest data appear to confirm there is a change in the frequency of HR-HPV occurrence, especially HPV-16 and HPV-18, as a reflection of the implementation of preventive vaccination programs. Owing to the growing incidence of cervical cancer among reproductive-age women and with the development of cancer management approaches, fertility-sparing options have been proposed for early-stage cervical cancer management as an option for young women, especially those with unaccomplished reproductive desires. However, methods applied for this purpose (cold-knife conization, loop electrosurgical excision, trachelectomy) have variable outcomes and do not prevent risks of relapse. Multiple factors are involved in cervical cancer recurrence, even in cases treated at the early stage of the disease. In this review, the authors unveil whether HPV infection and virus type could be one of the key factors associated with cervical cancer recurrence after fertility-sparing surgery. Reviews of the literature reveal that recurrent and persistent HR-HPV infection is a strong predictor of cervical lesions’ relapse. In particular, HPV-16 and HPV-18 infections and their persistence have been reported to be associated with cervical cancer recurrence. HR-HPV genotyping before and after fertility-sparing surgery for cervical cancer could facilitate a personalized approach and improve the overall survival rate. Screening for HR-HPV is essential during the follow-up of cervical cancer-treated women and will help to predict possible cancer recurrence.

## 1. Introduction

Cervical cancer is a malignant condition of the uterine cervix affecting women worldwide [1,2,3,4,5]. The causative agents and risk factors associated with cervical cancer are well-investigated [6,7]. The main etiological factor is human papillomavirus (HPV) infection, especially the high-risk (HR) types [6]. However, it should be noted that approximately 5% of cervical cancer cases are not associated with HPV infection [8].

Apart from HR-HPV infection, other factors may also play a role in the onset and development of cervical cancer. Such factors may include one or more of the following: the onset of sexual life before the age of 15; multiple sexual partners [9,10]; immunosuppressive conditions (e.g., HIV, long-term treatment with steroid hormones); cervical and vaginal microbiome alterations [11,12,13]; sexually transmitted infections (STIs) [14,15,16]; estrogen and progesterone imbalance, and sex-steroid hormone receptors malfunction [17,18,19,20]; genetic predisposition through polymorphism of HLA, MTHFR, PALB2, POLE3, as well as other possible genes and smoking [21,22,23].

Since the development and implementation of prophylactic HPV vaccination from 2006 onwards, an opportunity to prevent HPV infection dissemination and decrease the incidence of cervical cancer has become possible [24]. Currently, 136 countries worldwide have implemented HPV vaccination programs, which were originally recommended for girls aged 9–15 years old. However, recent study results suggest that the vaccination program can be useful for a wider range of age groups (i.e., females up to 45 years old) [24]. Moreover, HPV vaccination is increasingly recommended for patients diagnosed with precancerous cervical lesions and existing HPV infection as a complementary treatment [25]. Nevertheless, despite being one of the preventable cancers via HPV vaccination and cervical cancer screening programs, cervical cancer remains one of the top three cancers affecting females worldwide in the 35–45 age group [3,13,26].

Since many women are being diagnosed with cervical cancer in their reproductive age, before the completion of their fertility plans, modern gynecologic oncology is developing and implementing fertility-sparing approaches/guidelines to manage these young patients [27,28]. More recent sources have reported a success rate of fertility-preserving management of approximately 90% [29]. However, the risk of cervical cancer recurrence after fertility-sparing surgery still exists due to many factors [29,30,31]. In this review article, the authors disclose whether HPV infection, particularly infection with HR-HPV and other virus types, could be one of the critical factors associated with cervical cancer recurrence post-fertility-sparing surgery. This study’s hypothesis is that the presence of HPV infection and long-term persistence of the high-risk virus types result in higher recurrence post-fertility-sparing surgery.

## 2. Material and Methods

### 2.1. Literature Search

**Figure 1:**
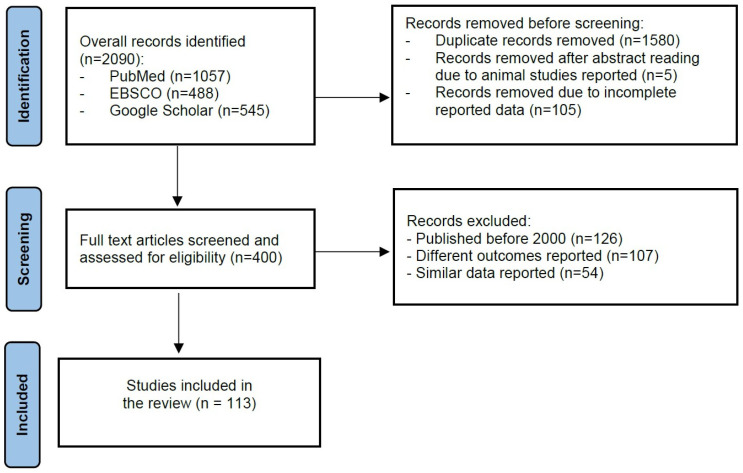
Data extraction flowchart.

Articles published in English were searched in PubMed/MEDLINE, Google Scholar, and EBSCO from January 2000 to September 2024. The search was performed using the following keywords: “early-stage cervical cancer”, “fertility-sparing”, “fertility preservation”, “fertility-sparing surgery”, “human papillomavirus”, “HPV”, “high-risk HPV”, “recurrence”, and “risk-factors”. Medical subject heading (MeSH) terms were used whenever available: “Uterine Cervical Neoplasms” (MeSH Unique ID D002583) as a major topic, “human papillomavirus” (MeSH Unique ID: D000094302), “fertility preservation” (MeSH Unique ID: D059247), and “E7 protein, HPV type 16” (MeSH Unique ID C059731). The search was specified and targeted by using “cervical cancer” OR “early-stage cervical cancer”, AND “fertility-sparing”, OR “fertility preservation”, OR “fertility-sparing surgery”, AND “recurrence”, AND “risk-factors”, AND “human papillomavirus”, OR “HPV”, AND/OR “high-risk HPV” (Figure 1).

### 2.2. Inclusion and Exclusion Criteria

Original studies, previously published reviews, and one case report were identified using the highlighted keywords, and keyword combinations were included in this comprehensive review article. Inclusion criteria: articles published in English from January 2000 to September 2024 (the literature search period) and fulfilling the keywords applied. Exclusion criteria: articles published in languages other than English prior to January 2000 and not meeting the keywords in the search strategy. Titles and abstracts of articles were retrieved by applying the search strategy and investigated individually by two review authors to categorize samples that could potentially meet the aims of this review. Duplicated studies and irrelevant articles that did not fulfill the listed search criteria were excluded. Full texts of these hypothetically eligible studies were retrieved (whenever available) and independently evaluated for suitability by the other two team members. Any discrepancy regarding the eligibility of specific papers was resolved through mutual discussion among the research team members. Peer-reviewed articles published in English and discussing HPV infection, HPV type, cervical cancer, and recurrence after fertility-sparing surgery were included in this review. Based on the content of this study’s findings and the heterogeneity of the articles, a narrative synthesis of the data was applied.

## 3. Results and Discussion

### 3.1. Epidemiology of Cervical Cancer

Cervical cancer remains a global public health issue [32]. It continues to appear as the fourth most common cancer among women worldwide and one of the primary causes of cancer-related mortality among females in the developing world [1,3,4,26,32,33]. The situation with cervical cancer incidence is more or less stable and under control in the developed world due to the impact of successful primary and secondary prevention programs [34,35,36]. However, low- and middle-income countries (LMICs) have a high prevalence and increasing incidence of the disease as a result of the inconsistent implementation of preventative measures [2,3,4,32,33,37].

The tendencies in cervical cancer incidence and mortality have remained similar for the past 10 years, with increasing proportions in LMICs [4,32,38,39]. In 2018, 569,000 new cervical cancer cases and 311,000 cervical cancer-related deaths were reported [2], while in 2020, there were more than 600,000 cervical cancer cases and almost 350,000 cervical cancer-related deaths occurred globally [32]. The global estimated age-standardized incidence of cervical cancer is 13.3 per 100,000 women/year, and the average age at death from cervical cancer is 59 years (age range 45–79 years) [4,32,33]. However, these indicators vary widely among countries, with the maximum incidence and mortality rates being registered in low-income countries [32]. Overall, 84% of new cervical cancer cases and up to 90% of the disease-related deaths occur in LMICs [2].

Based on the age-standardized incidence of the reports, cervical cancer ranks as the third most frequent cancer among women younger than 45 years in almost 80% of countries in the world [3]. Moreover, according to the recent International Agency for Research on Cancer (IARC) report, cervical cancer is the second most prevalent cancer and the second most common cause of cancer-related death among reproductive-age women (15–44 years) in 23 countries globally (mostly in sub-Saharan Africa) [3].

### 3.2. Cervical Cancer and Human Papillomavirus

Human papillomavirus is the most common sexually transmitted virus in the world; however, the non-sexual route of transmission is also considered important [37,40,41]. The lifetime risk of HPV contraction is estimated at 85% [42]. Fortunately, up to 80–90% of HPV infections are resolved by the host immune system without any clinical consequences [37].

Based on their cancerogenic properties, HPVs are divided into two groups—low-risk (LR) and high-risk (HR) HPV infections [43,44]. The high-risk cancerogenic HPV types include HPV-16, -18, -31, -33, -35, -39, -45, -51, -52, -56, -58, -59, -68, -73, and -82 [45]. Approximately 5% of all types of cancers worldwide are related to HPV infection [37,46]. The association between HPV infection and cervical cancer has been proven, and more than 95% of cervical cancer cases are associated with HR-HPV genotypes [6,37,40,43,46].

#### Prevalence of HR-HPV in Cervical Specimens

More than 35 HPV genotypes have been identified from anogenital neoplastic lesions [40,41,43]. Of all HPV-related cervical cancers, HPV-16 and HPV-18 types are responsible for approximately 70%, and the remaining HR-HPV types account for up to 25% [37]. The detailed analysis of the HR-HPV types’ distribution reveals that HPV-16 accounts for 50–60% and HPV-18—for 10–20% of cervical cancer cases in most countries [40]. Of the other most common HR-HPV types, HPV-45 and HPV-31 can be found in 4–8% and 1–5% of samples, respectively [40]. One of the recent sources reported the global frequency of the most prevalent HR-HPV genotypes detected in cervical cancer specimens are HPV-16 (up to 83. 8%) and HPV-18 (41%). The other most prevalent HR-HPV types include HPV-52 (40.7%), HPV-51 (18.8%), HPV-58 (15.6%), HPV-39 (13.3%), HPV-68 (11%), HPV-31, HPV-33, HPV-45, and HPV-56, with equal distribution of approximately 9%, HPV-59 (4.4%), and HPV-35 (3.2%) [47].

**Figure 2:**
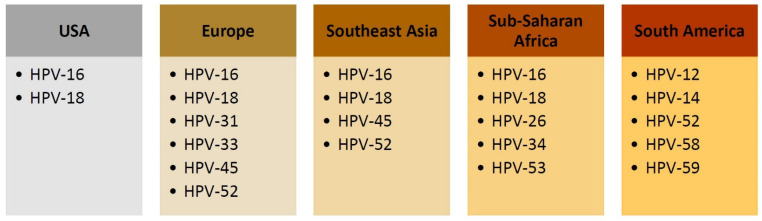
Most prevalent HPV types identified in cervical specimens.

The prevalence and distribution of the HPV types that are responsible for cervical carcinogenesis vary depending on the region (Figure 2). An earlier study from the UK identified HR-HPV genotypes in 87% of samples of microinvasive (stage IA) cervical cancer [48]. In the USA, the most prevalent HR-HPV types are HPV-16 and HPV-18 [37,49]. In Central and South American regions, HPV-12, HPV-14, HPV-52, HPV-58, and HPV-59 were the most prevalent, accounting for 15% to 25% of all HR-HPVs [37]. In a study from Sweden, the most common HR-HPV types identified in cervical cancer specimens were HPV-16 (60%), followed by HPV-18 (19%), HPV-45 (7%), and HPV-31 (3%), with HPV-33 and HPV-52 contributing equally (2%) [50]. The other HR-HPV types had an equal contribution of 1%, these being HPV-39, HPV-70, HPV-56, HPV-35, HPV-58, and HPV-59. In a study from Poland assessing the prevalence of specific HPV types among patients with high-grade squamous intraepithelial lesions (HSIL), HPV-16, HPV-31, HPV-52, HPV-66, HPV-53, and HPV-51 genotypes were the most prevalent [51].

A study from Southeast Asia found HR-HPV types in up to 97% of confirmed invasive cervical cancer and in 100% of adenocarcinoma in situ (AIS) cases [52]. The most prevalent HR-HPV types observed in patients with invasive cervical cancer were HPV-16 (61%), HPV-18 (35%), HPV-45 (17%), and HPV-52 (10%) [52]. A later study from the region reported that the most frequently detected HR-HPV types in cervical lesions are HPV-16, HPV-18, HPV-31, and HPV-33 [47]. The HR-HPV types and distribution among women with cervical cancer may differ depending on patients’ age and cancer stage [47].

A more recent study from Iran reported HR-HPV types identified among 78.8% of cervical cancer samples, including HPV-16 in 43.4% and HPV-18 in 8% of cases [53]. The average age of women with cervical cancer in the reported study was 53 years old. In a study from Botswana among patients with invasive cervical cancer, the most common HR-HPV types listed according to the frequency of identification were HPV-26, HPV-34, HPV-16, HPV-18, and HPV-53 [54].

The most recent large-scale study from China investigating the prevalence and characteristics of HPV among Chinese women with cervical lesions of various severity reported HPV-16 to be the most prevalent high-risk type (59.4%), followed by HPV-18 (22.2%), HPV-52 (7.7%), HPV-58 (7.3%), and HPV-33 (4.8%) [5]. Moreover, the authors highlighted that the magnitudes of non-HPV 16/18-attributed cervical cancers increased with the study participants’ age.

Interestingly, co-contamination with both high- and low-risk HPVs was reported to have reduced risk association with future invasive cervical cancer [55]. Thus, the researchers proposed that co-infection of LR-HPV and HR-HPV affects the probability of progression to invasive cervical cancer.

### 3.3. Fertility-Sparing Surgery for Women with Cervical Cancer

Many women are being diagnosed with reproductive organ cancers in their reproductive age [56,57]. According to the USA National Cancer Institute (NCI), nearly 40% of women diagnosed with cervical cancer are younger than 40 years [58,59,60], and based on the CDC reports, the maximum incidence of cervical cancer is among women aged 35 to 49 years [61,62]. Moreover, the maternal age at first pregnancy has been increasing over the recent decade [59,63]. Thus, fertility-sparing treatment is a crucially important management option, especially for those who have not completed their reproductive plan [31,58,64,65].

A standard of treatment for the early stages of cervical cancer implies a radical hysterectomy with lymph node assessment [60]. However, according to the most recent guideline from the European Society of Gynecological Oncology (ESGO) with the European Society for Radiotherapy and Oncology (ESTRO) and the European Society of Pathology (ESP), fertility-sparing surgery could be applied for early-stage cervical cancer patients (IB1, IB2, and IIA1 stages by Fédération Internationale de Gynécologie et d’Obstétrique (FIGO)) [27].

#### 3.3.1. Fertility-Sparing Surgical Procedures

Currently, several effective methods are available for fertility-sparing treatment of cervical cancer [61]. These include cold knife conization (CKC) with endocervical curettage, a loop electrosurgical excision procedure (LEEP), as well as simple and radical trachelectomy [61,65,66].

CKC with endocervical curettage may be considered for patients with cervical cancer at stage IA1 (FIGO) [61,62,65]. A recent report confirms progressively improving performance of CKC with lymph node assessment for early-stage cervical cancer (IB1, IB2, and IIA1) management [67]. The risk of treatment failure is lower in CKC compared to LEEP [68,69]. Thus, CKC is preferable to LEEP due to the possibility of missing margins during electro-excision surgery [61]. In women post-CKC, for early-stage cervical cancer, the five-year survival rate is reported at the level of 98–99% [61]. These data are in line with reports highlighting that there is “no difference in disease-specific survivals between patients treated with conization, trachelectomy, or hysterectomy” [58].

Simple trachelectomy, likewise CKC, is suggested as an acceptable fertility-sparing procedure in patients with IA1 and IA2 cervical cancer, “regardless of LVSI status” [27,61,69]. The procedure has a lower risk of complications compared to radical trachelectomy [60,65] and less subsequent obstetric morbidity [58]. Particularly, the live-birth rate was higher post-CKC and simple trachelectomy compared to abdominal radical trachelectomy (86.4% and 65.7%, respectively) [58].

Radical trachelectomy implies the removal of the uterine cervix with surrounding parametrial tissues and can be performed via laparotomic (abdominal) and vaginal approaches or minimally invasive surgery [61,66]. Considering the fertility-sparing purpose of this procedure, the proximal 5 mm of the cervix should be left in place in order to allow cervical cerclage later if pregnancy is achieved [61].

According to the recent ESGO/ESTRO/ESP guidelines, radical trachelectomy is the standard of treatment for patients who desire fertility-sparing surgery with cervical cancer stage IA1, IA2, or IB1 because the recurrence rate is similar to those after radical hysterectomy and ranges between 95% to 100% [27,61]. However, patients with tumors larger than 2 cm are not appropriate candidates for radical trachelectomy due to the increased risk of recurrence [58,70]. Moreover, a radical trachelectomy itself may lead to further conception- and pregnancy-related complications [60]. Thus, fertility-sparing treatment should be considered for appropriately selected candidates after assessment of their fertility potential [69].

Another recent study included patients with tumors ≤ 2 cm and depths of stromal invasion < 10 mm with negative nodes and who were treated by a simple hysterectomy [71,72]. In this study, patients with early-stage cervical cancer were reported to have a similar outcome compared with patients where a radical hysterectomy was performed [71,72]. This definitely appears to have an impact on the fertility-sparing treatment of patients with a tumor size ≤ 2 cm and depths of stromal invasion < 10 mm.

While considering the fertility-sparing approach for cervical cancer, the possibility of the disease recurrence has to be kept in consideration [27].

#### 3.3.2. Success Rates and Outcomes of Fertility-Sparing Surgery

The success rates of fertility-sparing surgery for cervical cancer can be measured by live-birth rate, rates of obstetric complications, and cervical cancer recurrence rate. To date, there is evidence that, for many patients, fertility-sparing treatments have resulted in reproductive function preservation and improved obstetrical outcomes “without compromising oncologic safety” [73,74,75].

Recent research reported excellent obstetric outcomes after fertility-sparing surgery [58,60,76]. These studies identified fertility rates of 55% after fertility-sparing treatments [59], while the pregnancy rates after vaginal, abdominal, and laparoscopic radical trachelectomies were 37.8%, 10.4%, and 9.2%, respectively [58].

The overall live-birth rate after fertility-sparing surgery is reported as 70% [60]. The live-birth rate was higher post-CKC, and simple trachelectomy compared to abdominal radical trachelectomy yielded results of 86.4% and 65.7%, respectively [58]. However, the success rate is lower after vaginal and minimally invasive surgery.

The overall preterm birth rates after fertility-sparing surgeries range between 31 and 38% [58,60], including the lowest rate post-CKC or simple trachelectomy (25%), and the highest rate after vaginal trachelectomy (34.6%) [58]. Infertility rates after fertility-sparing surgery range between 14% and 41% [60]. One of the major causes of infertility (with a rate of 33%) after fertility-sparing surgery is cervical stenosis [60,77].

Many women require assisted reproductive technologies (ART) treatment after fertility-sparing management [60,75]. The ART-related pregnancy rate after fertility-sparing surgery for cervical cancer ranges between 13% and 67%, with a live-birth rate of 16–100% [78]. Preterm labor rates after ART are higher than in spontaneous pregnancy after fertility-sparing surgery and reach 50% [78,79]

Overall, gestations after fertility-sparing surgery appear with a higher risk of obstetric complication (preterm premature rupture of membranes and preterm birth) [58,79,80].

### 3.4. Cervical Cancer Recurrence After Fertility-Sparing Management

Cervical cancer recurrence is the relapse of the cervical tumor more than six months after the end of treatment and complete regression of the tumor [30,81,82]. It usually appears within 2 years after the initial treatment [82]; however, the longest timeframe of cervical cancer recurrence after non-radical surgery reported to date was recorded as 18 years, and the case was associated with HR-HPV-positive cancer [83].

Recurrence of cervical cancer occurs mainly within 3 years after the initial surgery, while late recurrence is rare (0.8–4%) [84]. The estimated recurrence rate in patients with tumors ˂ 2 cm following surgery is reported to be around 1.2% [82]. However, with an increase in the tumor stage and size, the chance of recurrence likewise increases [82]. According to recent reports, the recurrence rates of early-stage cervical cancer (stage IB and IIA, FIGO) are approximately 10% and 17%, respectively [82,84], while in later stages, the recurrence after surgery (IIB, III, and IVA, FIGO) has been estimated at 23%, 42%, and 74%, respectively [84].

#### 3.4.1. Cervical Cancer Recurrence After Fertility-Sparing Surgery

The problem of cervical cancer recurrence after fertility-sparing surgery has been investigated in many studies; however, mostly with a relatively low or limited sample size [66,78,85,86].

Earlier studies on the outcomes of fertility-sparing surgeries for early-stage cervical cancer (IA1-IB1, FIGO) reported a recurrence rate of 2.9% for tumor size ˂ 2 cm and a high risk of cancer relapse in cases with tumor size of ˃2 cm (up to 20.8%) [69,85].

In a study that focused on recurrence rates in stages IB1 and IB2 of cervical cancer post- CKC, simple and radical trachelectomy, the lowest rate of recurrence is observed in women undergoing abdominal radical trachelectomy—2.4% [66]. The recurrence post-CKC, simple trachelectomy, and radical laparoscopic trachelectomy were 4.1%, 4.7%, and 5.2%, respectively [66].

A study of the outcomes after vaginal radical trachelectomy for early-stage cervical cancer with most of the participants at stage IB1 resulted in a 6.8% recurrence rate [87]. The researchers reported that the non-squamous cell histological type of cervical carcinoma and high-grade disease were associated with a “significantly higher risk of recurrence” [85,86,87].

A systematic review of fertility-sparing surgery outcomes in gynecologic cancers reported a significantly higher recurrence in women with stage IB1 and IB2 disease than in women with stage IA1 and IA2, at 3.1% and 5.6% vs. 0.2% and 0.7%, respectively [88]. The cervical cancer recurrence rate after fertility-sparing surgery and subsequent ART was reported to be 3.9% and “comparable to the outcomes after radical hysterectomy” [78,88].

Thus, based on the studies analyzed, it is clear that the recurrence rate after fertility-sparing surgery largely depends on the cancer stage and size, and, therefore, appropriate and accurate patient selection may help to improve fertility-sparing surgery outcomes.

#### 3.4.2. Cervical Cancer Recurrence After Neoadjuvant Chemotherapy and Fertility-Sparing Surgery

Currently, researchers suggest combining neoadjuvant chemotherapy (NACT) with fertility-sparing surgery as an alternative management to the standard treatment of cervical cancers >2 cm. However, the efficacy and validity of the suggested approach remain a topic for the current debate concerning safety in relation to future pregnancy [89].

A retrospective study of women with cervical cancer from 2 to 6 cm who received NACT prior to abdominal radical trachelectomy demonstrated that this option may be reasonable and safe only in selected patients with cervical cancer >2 cm [89]. Another retrospective study, which explored the optimum fertility-sparing treatment for early-stage cervical cancer (stage IB2, FIGO), reported the results of treatment for patients who have undergone NACT with radical trachelectomy [90]. The researchers concluded that NACT followed by radical trachelectomy could be “a feasible fertility-sparing option for selected patients with 1B2 cervical cancer” [90].

A systematic review of oncological outcomes after fertility-sparing management for early-stage cervical cancer revealed significant heterogeneity in clinical management [91]. According to the mentioned study, considering the oncological outcomes, treatment techniques limited to minimally invasive or vaginal surgery exhibited the highest recurrence rate. Another systematic review on this field, which assessed the oncologic and fertility outcomes of patients with cervical cancer >4 cm of women post-NACT followed by fertility-sparing surgery, reported a complete pathological response in 56% [92]. In this study, the recurrence occurred in 7.7% of cases. However, according to the cited authors, evidence supporting the application of fertility-sparing surgery post-r NACT in patients with cervical cancer >4 cm is limited.

A recently published paper highlighted the point that for patients with cervical tumors > 2 cm and histopathologically cancer-free lymph nodes, NACT and radical vaginal trachelectomy could be applied to women planning pregnancy [86]. In the cited study, the pregnancy rate resulting in healthy newborns was 55%. The authors underlined the fact that this fertility-sparing tactic is associated with higher relapse and mortality compared with previously available literature for patients undergoing radical vaginal trachelectomy for a tumor size of ˂2 cm. Considering the fact that insufficient data are available, patients with cervical cancer >2 cm and histopathologically tumor-free lymph nodes should not be offered the mentioned approach on a routine basis [86]. Thus, due to the unavailable standards for NACT for patients with early-stage cervical cancer with future fertility plans and heterogeneous results of the existing study results, this method should be considered as a research intervention and requires further investigations with a larger sample size.

However, another recent research reported the importance of fertility-sparing treatment for young women with early-stage cervical cancer when the tumor size is >2 cm [69]. This study suggests that “fertility-sparing approaches hold promise for preserving reproductive function” among young women diagnosed with early-stage cervical cancer [69]. Nevertheless, the authors highlighted that more studies with a long-term follow-up are required to evaluate the “oncologic safety and fertility preservation efficacy” of contemporary fertility-sparing approaches.

### 3.5. Risk Factors for Cervical Cancer Recurrence After Fertility-Sparing Management

**Figure 3:**
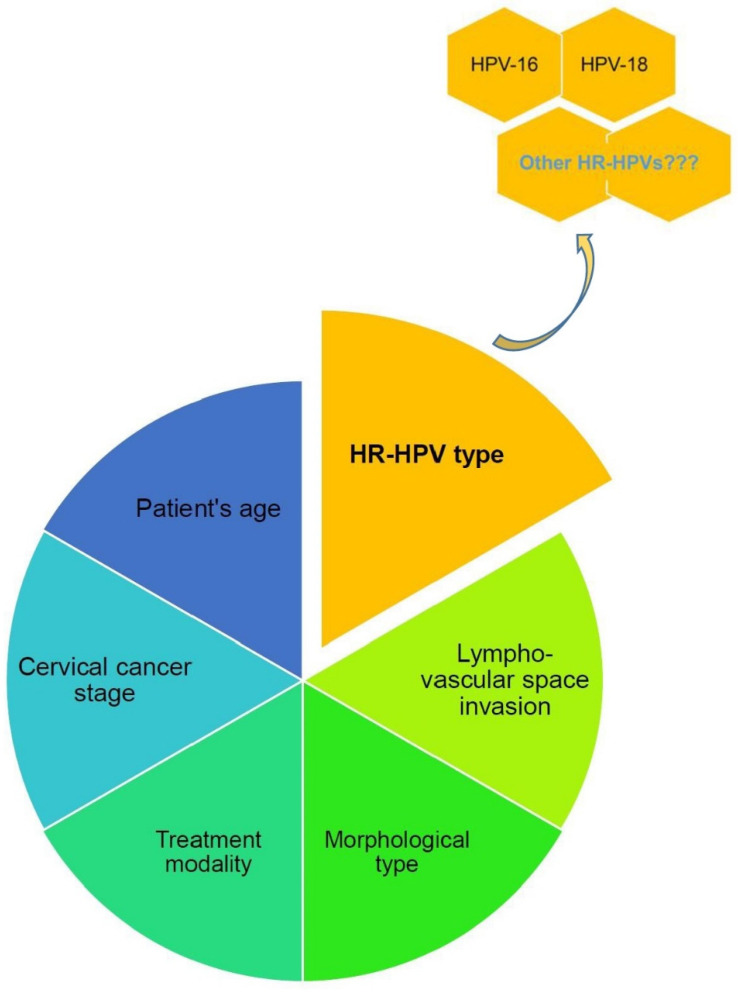
Factors related to cervical cancer recurrence.

Multiple factors play a role in cervical cancer recurrence. These are cervical cancer stage, morphological type (adenocarcinoma or squamous cell carcinoma), lympho-vascular space invasion, type of treatment, HPV persistence, patient’s age, etc. (Figure 3) [30,31,82,93].

The most recent study investigating risks of cervical cancer recurrence after fertility-sparing treatment reported that “fertility-sparing cervical procedures were not associated with an increased risk of recurrence compared with radical procedures in patients with tumors ≤ 2 cm in size” [31]. Studies among women with early-stage cervical cancer and a tumor size ≤ 2 cm report similar oncological outcomes in women who had radical cervical surgery and those who underwent fertility-sparing surgery [31,94,95]. Similar findings were received by the recent SHAPE study [71,72]. Moreover, researchers reported that parametrectomy, which may later negatively impact perinatal outcomes, did not result in a better prognosis in women with stage IB1 cervical cancer [31]. Fertility-sparing management, “regardless of their radicality, were associated with substantially worse oncologic outcomes” in women with stages IB2 and higher by FIGO. Lympho-vascular space invasion is another key risk factor used for the prediction of cervical cancer recurrence risk [30,31].

The results of the FERTISS study representing the largest cohort after fertility-sparing surgery for early-stage cervical cancer have confirmed that the oncological outcomes after non-radical treatment depend on the tumor size [30]. In this study, women after fertility-sparing surgery with a tumor size ≤ 2 cm had excellent oncological outcomes “in patients with HPV-associated tumors” without lymph nodes involvement, while in women with tumors > 2 cm in size “a significantly higher risk of recurrence, regardless of the tumor type” was found [31].

Other than the cancer stage, size, and patient age, there are other individual risk factors for cervical cancer recurrence. Obesity, smoking, and excessive alcohol intake after fertility-sparing surgery also increase the risk of recurrence [82].

### 3.6. Role of HPV Type in Cervical Cancer Recurrence After Fertility-Sparing Surgery

Whilst the contributing factors leading to cervical cancer recurrence are not fully proven, what is clear is that the research data show that patients with cervical cancer recurrence have a worse prognosis and high mortality rates [82].

The fact that women vaccinated with one of the available HPV vaccines at the time of surgical treatment for high-grade cervical lesions had a lower risk of recurrence compared with unvaccinated controls confirms the definitive role of HPV infection in cervical cancer recurrence [93,94,96,97]. The HPV types responsible for the infection recurrence could be identified by the same methods as used for initial HPV infection: PCR genotyping or mRNA tests. Moreover, circulating HPV DNA was reported as a useful marker to predict the recurrence of cervical cancer [98,99]. One of the recent studies suggests that young women ˂35 years old with cervical lesions and p16-positive immunohistochemistry tests had a worse “post-excisional course” prognosis after the “cervix-sparing” procedure compared with p16-negative controls [98].

An earlier study concluded that patients with HPV-negative cervical cancer had a significantly worse prognosis after radiotherapy. They suggested the use of HPV status as a marker for the optimization of cervical cancer management [100,101]. A later systematic review reported that HPV detection in lymph nodes may improve the “accuracy of micro-metastasis detection”, thus helping women with a high risk of cervical cancer recurrence and allowing the identification of appropriate patients for fertility-sparing treatment [102].

A Norwegian study on the role of specific HPV types in the recurrence of cervical lesions concluded that high-grade cervical lesions were associated with persistent HPV-16 and HPV-18 in patients undergoing CKC [103]. This was confirmed by other studies evaluating the risk factors for relapse of high-grade cervical lesions or cervical cancer in situ, which determined that postoperative persistent HPV-16 and HPV-18 infections cause recurrence of high-grade cervical lesions and, therefore, serve as a potential risk factor for cervical cancer recurrence [93,104,105,106,107,108,109,110] Another study exploring risk factors for cervical cancer recurrence reported that patients with persistent HPV-16 infection after cervical excision were at a particularly high risk of cancer relapse and progression [111]. Thus, according to the available evidence, persistent HPV-16 and HPV-18 in women undergoing CKC can serve as a risk factor for cervical cancer recurrence.

Moreover, HPV testing and colposcopy revealed the highest sensitivity for the detection of cervical cancer recurrence after fertility-sparing surgery [29]. Thus, HR-HPV testing plays an essential role in the follow-up of women after fertility-sparing surgery for early-stage cervical cancer [48,94,98,103,105]. Moreover, research evidence shows that HPV vaccination reduces the risk of cervical lesion recurrence in patients after surgically treated high-grade cervical lesions (precancer) [65,112,113].

Study strengths and limitations. The main strength of this study is based on the comprehensive review of up-to-date information related to HR-HPV infection and cervical lesion recurrence after initial management. The authors investigated literature covering the past 24 years to validate the hypothesis of the study. However, some limitations should be taken into account: (1) only papers written in the English language were included; (2) a narrative, non-systematic synthesis was performed due to the heterogeneity of studies identified in the existing literature; (3) insufficient data were found on HR-HPV types that are the most frequently associated with the recurrence with the link to the type of fertility-sparing surgery in women with early-stage cervical cancer.

Future research implications. Since the findings of researchers on the role of the HR-HPV type in cervical lesion recurrence are controversial [108,109], more studies with large sample sizes investigating factors of cervical cancer recurrence after fertility-spring surgery and specifically focusing on the role of HR-HPVs are required to shed more light on the causes of the disease relapse [95].

Clinical implications. Identifying certain HR-HPV infection types responsible for cervical lesions’ recurrence after fertility-sparing treatment could improve the potential preventive measures used after treatment. As highlighted by previous studies, up to 8% of women after fertility-sparing treatment of precancerous cervical lesions may experience a recurrence of the condition [65,113]. Understanding the HPV types responsible for cervical lesion recurrence might assist in helping to choose the appropriate prophylactic HPV vaccine, which could be used “before or after surgical management of premalignant cervical lesions” and thus reduce the risk of relapse [65].

## 4. Conclusions

Fertility-sparing surgery for cervical cancer management has been proven as a valid and reliable approach in young women. Persistent HR-HPV infection is a strong predictor of disease relapse. In particular, HPV-16 and HPV-18 infections and their persistence were reported to be associated with cervical cancer recurrence. HR-HPV genotyping before and after fertility-sparing surgery for cervical cancer could facilitate a personalized approach and may improve the survival rate. Long-lasting follow-up studies utilizing HR-HPV genotyping and involving patients with cervical cancer recurrence after fertility-sparing surgery will help in a better understanding of the HR-HPV role in cervical cancer recurrence and identify the most common types associated with disease relapse. Thus, screening for HR-HPV is essential during the follow-up of cervical cancer-treated women and will help to predict the likelihood of cancer recurrence.

## References

[1] Klein C., Kahesa C., Mwaiselage J., West J.T., Wood C., Angeletti P.C. (2020). How the Cervical Microbiota Contributes to Cervical Cancer Risk in Sub-Saharan Africa. Front. Cell Infect. Microbiol..

[2] Hull R., Mbele M., Makhafola T., Hicks C., Wang S., Reis R.M., Mehrotra R., Mkhize-Kwitshana Z., Kibiki G., Bates D.O. (2020). Cervical cancer in low and middle-income countries. Oncol. Lett..

[3] Arbyn M., Weiderpass E., Bruni L., de Sanjosé S., Saraiya M., Ferlay J., Bray F. (2020). Estimates of incidence and mortality of cervical cancer in 2018: A worldwide analysis. Lancet Glob. Health.

[4] Bruni L., Albero G., Serrano B., Mena M., Collado J.J., Gómez D., Muñoz J., Bosch F.X., de Sanjosé S. ICO/IARC Information Centre on HPV and Cancer (HPV Information Centre). Human Papillomavirus and Related Diseases in the World. Summary Report 10 March 2023. https://hpvcentre.net/statistics/reports/XWX.pdf.

[5] Huiyun J., Jie D., Huan W., Yuebo Y., Xiaomao L. (2023). Prevalence and characteristics of cervical human papillomavirus genotypes and cervical lesions among 58,630 women from Guangzhou, China. J. Infect. Public Health.

[6] zur Hausen H. (2009). Papillomaviruses in the causation of human cancers—A brief historical account. Virology.

[7] Araldi R.P., Sant’Ana T.A., Módolo D.G., de Melo T.C., Spadacci-Morena D.D., de Cassia Stocco R., Cerutti J.M., de Souza E.B. (2018). The human papillomavirus (HPV)-related cancer biology: An overview. Biomed. Pharmacother..

[8] Cancer Genome Atlas Research Network, Albert Einstein College of Medicine, Analytical Biological Services (2017). Integrated genomic and molecular characterization of cervical cancer. Nature.

[9] Park Y., Baik S., Ho C., Lin C.Y., Chung S.H. (2021). Progesterone Receptor Is a Haploinsufficient Tumor-Suppressor Gene in Cervical Cancer. Mol. Cancer Res..

[10] Bowden S.J., Bodinier B., Kalliala I., Zuber V., Vuckovic D., Doulgeraki T., Whitaker M.D., Wielscher M., Cartwright R., Tsilidis K.K. (2021). Genetic variation in cervical preinvasive and invasive disease: A genome-wide association study. Lancet Oncol..

[11] Ntuli L., Mtshali A., Mzobe G., Liebenberg L.J., Ngcapu S. (2022). Role of Immunity and Vaginal Microbiome in Clearance and Persistence of Human Papillomavirus Infection. Front. Cell Infect. Microbiol..

[12] Frąszczak K., Barczyński B., Kondracka A. (2022). Does Lactobacillus Exert a Protective Effect on the Development of Cervical and Endometrial Cancer in Women?. Cancers.

[13] Kyrgiou M., Moscicki A.B. (2022). Vaginal microbiome and cervical cancer. Semin. Cancer Biol..

[14] Fracella M., Oliveto G., Sorrentino L., Roberto P., Cinti L., Viscido A., Di Lella F.M., Giuffrè F., Gentile M., Pietropaolo V. (2022). Common Microbial Genital Infections and Their Impact on the Innate Immune Response to HPV in Cervical Cells. Pathogens.

[15] Fazlollahpour-Naghibi A., Bagheri K., Almukhtar M., Taha S.R., Zadeh M.S., Moghadam K.B., Tadi M.J., Rouholamin S., Razavi M., Sepidarkish M. (2023). Trichomonas vaginalis infection and risk of cervical neoplasia: A systematic review and meta-analysis. PLoS ONE.

[16] Hamar B., Teutsch B., Hoffmann E., Hegyi P., Váradi A., Nyirády P., Hunka Z., Ács N., Lintner B., Hermánné R.J. (2023). Trichomonas vaginalis infection is associated with increased risk of cervical carcinogenesis: A systematic review and meta-analysis of 470,000 patients [published online ahead of print, 2023 Apr 3]. Int. J. Gynaecol. Obstet..

[17] Baik S., Mehta F.F., Unsal E., Park Y., Chung S.H. (2022). Estrogen Inhibits Epithelial Progesterone Receptor-Dependent Progestin Therapy Efficacy in a Mouse Model of Cervical Cancer. Am. J. Pathol..

[18] Asthana S., Busa V., Labani S. (2020). Oral contraceptives use and risk of cervical cancer-A systematic review & meta-analysis. Eur. J. Obstet. Gynecol. Reprod. Biol..

[19] Gadducci A., Cosio S., Fruzzetti F. (2020). Estro-progestin Contraceptives and Risk of Cervical Cancer: A Debated Issue. Anticancer. Res..

[20] Kamani M., Akgor U., Gültekin M. (2022). Review of the literature on combined oral contraceptives and cancer. Ecancermedicalscience.

[21] Tu S., Zhang H., Yang X., Wen W., Song K., Yu X., Qu X. (2021). Screening of cervical cancer-related hub genes based on comprehensive bioinformatics analysis. Cancer Biomark..

[22] Gong J.M., Shen Y., Shan W.W., He Y.X. (2018). The association between MTHFR polymorphism and cervical cancer. Sci. Rep..

[23] Shim H., Park B., Shin H.-J., Joo J., Yoon K.-A., Kim Y.M., Hayashi T., Tokunaga K., Kong S.-Y., Kim J.-Y. (2019). Protective association of HLA-DRB1*13:02, HLA-DRB1*04:06, and HLA-DQB1*06:04 alleles with cervical cancer in a Korean population. Hum. Immunol..

[24] Akhatova A., Azizan A., Atageldiyeva K., Ashimkhanova A., Marat A., Iztleuov Y., Suleimenova A., Shamkeeva S., Aimagambetova G. (2022). Prophylactic Human Papillomavirus Vaccination: From the Origin to the Current State. Vaccines.

[25] Pruski D., Millert-Kalińska S., Łagiedo M., Sikora J., Jach R., Przybylski M. (2023). Effect of HPV Vaccination on Virus Disappearance in Cervical Samples of a Cohort of HPV-Positive Polish Patients. J. Clin. Med..

[26] Ferrall L., Lin K.Y., Roden R.B.S., Hung C.F., Wu T.C. (2021). Cervical Cancer Immunotherapy: Facts and Hopes. Clin. Cancer Res..

[27] Cibula D., Raspollini M.R., Planchamp F., Centeno C., Chargari C., Felix A., Fischerová D., Jahnn-Kuch D., Joly F., Kohler C. (2023). ESGO/ESTRO/ESP Guidelines for the management of patients with cervical cancer—Update 2023. Int. J. Gynecol. Cancer.

[28] Koh W.J., Abu-Rustum N.R., Bean S., Bradley K., Campos S.M., Cho K.R., Chon H.S., Chu C., Clark R., Cohn D. (2019). Cervical Cancer, Version 3.2019, NCCN Clinical Practice Guidelines in Oncology. J. Natl. Compr. Cancer Netw..

[29] Wolswinkel J.T., Eikelder M.L.G.T., Verhoef C.G., Zusterzeel P.L.M. (2023). High- or Intermediate-Risk Histologic Features in Patients with Clinical Early-Stage Cervical Cancer Planned for Fertility-Sparing Surgery: A Systematic Review. Cancers.

[30] Moreira A.S.L., Cunha T.M., Esteves S. (2020). Cervical cancer recurrence-can we predict the type of recurrence?. Diagn. Interv. Radiol..

[31] Slama J., Runnebaum I.B., Scambia G., Angeles M.A., Bahrehmand K., Kommoss S., Fagotti A., Narducci F., Matylevich O., Holly J. (2023). Analysis of risk factors for recurrence in cervical cancer patients after fertility-sparing treatment: The FERTIlity Sparing Surgery retrospective multicenter study. Am. J. Obstet. Gynecol..

[32] Singh D., Vignat J., Lorenzoni V., Eslahi M., Ginsburg O., Lauby-Secretan B., Arbyn M., Basu P., Bray F., Vaccarella S. (2023). Global estimates of incidence and mortality of cervical cancer in 2020: A baseline analysis of the WHO Global Cervical Cancer Elimination Initiative. Lancet Glob. Health.

[33] Buskwofie A., David-West G., Clare C.A. (2020). A Review of Cervical Cancer: Incidence and Disparities. J. Natl. Med. Assoc..

[34] Issanov A., Karim M.E., Aimagambetova G., Dummer T.J.B. (2022). Does Vaccination Protect against Human Papillomavirus-Related Cancers? Preliminary Findings from the United States National Health and Nutrition Examination Survey (2011–2018). Vaccines.

[35] Simms K.T., Steinberg J., Caruana M., Smith M.A., Lew J.-B., Soerjomataram I., Castle P.E., Bray F., Canfell K. (2019). Impact of scaled up human papillomavirus vaccination and cervical screening and the potential for global elimination of cervical cancer in 181 countries, 2020–2099: A modelling study. Lancet Oncol..

[36] Vaccarella S., Franceschi S., Engholm G., Lönnberg S., Khan S., Bray F. (2014). 50 years of screening in the Nordic countries: Quantifying the effects on cervical cancer incidence. Br. J. Cancer..

[37] Petersen L.M., Fenton J.M., Kennedy L.S., LaRochelle E.P.M., Bejarano S., Tsongalis G.J. (2020). HPV, vaccines, and cervical cancer in a low- and middle-income country. Curr. Probl. Cancer.

[38] Aimagambetova G., Azizan A. (2018). Epidemiology of HPV Infection and HPV-Related Cancers in Kazakhstan: A Review. Asian Pac. J. Cancer Prev..

[39] Aimagambetova G., Chan C.K., Ukybassova T., Imankulova B., Balykov A., Kongrtay K., Azizan A. (2021). Cervical cancer screening and prevention in Kazakhstan and Central Asia. J. Med. Screen..

[40] Bosch F.X., de Sanjosé S. (2007). The epidemiology of human papillomavirus infection and cervical cancer. Dis. Markers.

[41] Bosch F.X., Qiao Y.L., Castellsagué X. (2006). CHAPTER 2 The epidemiology of human papillomavirus infection and its association with cervical cancer. Int. J. Gynaecol. Obstet..

[42] Centers for Disease Control and Prevention. https://www.cdc.gov/hpv/index.html.

[43] Castellsagué X. (2008). Natural history and epidemiology of HPV infection and cervical cancer. Gynecol. Oncol..

[44] Burd E.M., Dean C.L. (2016). Human Papillomavirus. Microbiol. Spectr..

[45] Okunade K.S. (2020). Human papillomavirus and cervical cancer. J. Obstet. Gynaecol..

[46] Yuan Y., Cai X., Shen F., Ma F. (2021). HPV post-infection microenvironment and cervical cancer. Cancer Lett..

[47] Xia C., Li S., Long T., Chen Z., Chan P.K.S., Boon S.S. (2021). Current Updates on Cancer-Causing Types of Human Papillomaviruses (HPVs) in East, Southeast, and South Asia. Cancers.

[48] Cairns M., Cuschieri K.S., Cubie H.A., Cruickshank M.E. (2010). High-risk HPV genotyping in the follow-up of women treated conservatively for microinvasive cervical cancer. Int. J. Gynecol. Cancer..

[49] Chaturvedi A.K., Graubard B.I., Broutian T., Xiao W., Pickard R.K.L., Kahle L., Gillison M.L. (2019). Prevalence of Oral HPV Infection in Unvaccinated Men and Women in the United States, 2009–2016. JAMA.

[50] Lagheden C., Eklund C., Lamin H., Kleppe S.N., Lei J., Elfström K.M., Sundström K., Andrae B., Sparén P., Dillner J. (2018). Nationwide comprehensive human papillomavirus (HPV) genotyping of invasive cervical cancer. Br. J. Cancer..

[51] Przybylski M., Pruski D., Wszołek K., de Mezer M., Żurawski J., Jach R., Millert-Kalińska S. (2023). Prevalence of HPV and Assessing Type-Specific HPV Testing in Cervical High-Grade Squamous Intraepithelial Lesions in Poland. Pathogens.

[52] Quek S.C., Lim B.K., Domingo E., Soon R., Park J.-S., Vu T.N., Tay E.H., Le Q.T., Kim Y.-T., Vu B.Q. (2013). Human papillomavirus type distribution in invasive cervical cancer and high-grade cervical intraepithelial neoplasia across 5 countries in Asia. Int. J. Gynecol. Cancer.

[53] Farahmand Z., Soleimanjahi H., Garshasbi M., Hasanzadeh M., Zafari E. (2021). Distribution of the most common types of HPV in Iranian women with and without cervical cancer. Women Health.

[54] Grover S., Seckar T., Gao L., Bhatia R., Lin X., Zetola N., Ramogola-Masire D., Robertson E. (2023). Characterization of HPV subtypes in invasive cervical cancer in Botswana patients using a pan-pathogen microarray technology. Tumour Virus Res..

[55] Sundström K., Ploner A., Arnheim-Dahlström L., Eloranta S., Palmgren J., Adami H.-O., Helm N.Y., Sparén P., Dillner J. (2015). Interactions Between High- and Low-Risk HPV Types Reduce the Risk of Squamous Cervical Cancer. J. Natl. Cancer Inst..

[56] Aimagambetova G., Terzic S., Laganà A.S., Bapayeva G., la Fleur P., Terzic M. (2021). Contemporary Fertility-Sparing Management Options of Early Stage Endometrioid Endometrial Cancer in Young Nulliparous Patients. J. Clin. Med..

[57] IARC (2022). Cervical Cancer Screening. IARC Handbooks of Cancer Prevention.

[58] Zaccarini F., Sanson C., Maulard A., Schérier S., Leary A., Pautier P., Chargari C., Genestie C., Gouy S., Morice P. (2021). Cervical Cancer and Fertility-Sparing Treatment. J. Clin. Med..

[59] Gwacham N.I., McKenzie N.D., Fitzgerald E.R., Ahmad S., Holloway R.W. (2021). Neoadjuvant chemotherapy followed by fertility sparing surgery in cervical cancers size 2–4 cm; emerging data and future perspectives. Gynecol. Oncol..

[60] Stewart K., Campbell S., Frumovitz M., Ramirez P.T., McKenzie L.J. (2021). Fertility considerations prior to conservative management of gynecologic cancers. Int. J. Gynecol. Cancer.

[61] Kohn J.R., Katebi Kashi P., Acosta-Torres S., Beavis A.L., Christianson M.S. (2021). Fertility-sparing Surgery for Patients with Cervical, Endometrial, and Ovarian Cancers. J. Minim. Invasive Gynecol..

[62] Nezhat C., Roman R.A., Rambhatla A., Nezhat F. (2020). Reproductive and oncologic outcomes after fertility-sparing surgery for early stage cervical cancer: A systematic review. Fertil. Steril..

[63] Sarría-Santamera A., Bapayeva G., Utepova G., Krstic J., Terzic S., Aimagambetova G., Shauyen F., Terzic M. (2020). Women’s Knowledge and Awareness of the Effect of Age on Fertility in Kazakhstan. Sexes.

[64] Silvestris E., Paradiso A.V., Minoia C., Daniele A., Cormio G., Tinelli R., D’Oronzo S., Cafforio P., Loizzi V., Dellino M. (2022). Fertility preservation techniques in cervical carcinoma. Medicine.

[65] Terzic M., Makhadiyeva D., Bila J., Andjic M., Dotlic J., Aimagambetova G., Sarria-Santamera A., Laganà A.S., Chiantera V., Vukovic I. (2023). Reproductive and Obstetric Outcomes after Fertility-Sparing Treatments for Cervical Cancer: Current Approach and Future Directions. J. Clin. Med..

[66] Morice P., Maulard A., Scherier S., Sanson C., Zarokian J., Zaccarini F., Espenel S., Pautier P., Leary A., Genestie C. (2022). Oncologic results of fertility sparing surgery of cervical cancer: An updated systematic review. Gynecol. Oncol..

[67] Furey K.B., Anderson Z.S., Kuznicki M.L., Klar M., Roman L.D., Wright J.D., Matsuo K. (2023). Increasing trends of cervical conization with lymph node evaluation for fertility-sparing surgery in early cervical cancer. Gynecol. Oncol..

[68] Athanasiou A., Veroniki A.A., Efthimiou O., Kalliala I., Naci H., Bowden S., Paraskevaidi M., Arbyn M., Lyons D., Martin-Hirsch P. (2022). Comparative effectiveness and risk of preterm birth of local treatments for cervical intraepithelial neoplasia and stage IA1 cervical cancer: A systematic review and network meta-analysis. Lancet Oncol..

[69] D’Amato A., Riemma G., Agrifoglio V., Chiantera V., Laganà A.S., Mikuš M., Dellino M., Maglione A., Faioli R., Giannini A. (2024). Reproductive Outcomes in Young Women with Early-Stage Cervical Cancer Greater than 2 cm Undergoing Fertility-Sparing Treatment: A Systematic Review. Medicina.

[70] Floyd J.L., Campbell S., Rauh-Hain J.A., Woodard T. (2021). Fertility preservation in women with early-stage gynecologic cancer: Optimizing oncologic and reproductive outcomes. Int. J. Gynecol. Cancer..

[71] Plante M., Kwon J.S., Ferguson S., Samouëlian V., Ferron G., Maulard A., de Kroon C., Van Driel W., Tidy J., Williamson K. (2024). Simple versus Radical Hysterectomy in Women with Low-Risk Cervical Cancer. N. Engl. J. Med..

[72] Takekuma M. (2024). Challenges and perspectives on less invasive surgery for early-stage cervical cancer: A critical analysis of the SHAPE trial and its implications. J. Gynecol. Oncol..

[73] Willows K., Lennox G., Covens A. (2016). Fertility-sparing management in cervical cancer: Balancing oncologic outcomes with reproductive success. Gynecol. Oncol. Res. Pract..

[74] Šimják P., Cibula D., Pařízek A., Sláma J. (2020). Management of pregnancy after fertility-sparing surgery for cervical cancer. Acta Obstet. Gynecol. Scand..

[75] Piątek S., Szymusik I., Bidziński M. (2023). Reproductive Results in Cancer Survivors after Fertility Sparing Management: The Need for the Standardization of Definitions. Cancers.

[76] Robova H., Rob L., Halaska M.J., Drozenova J., Pichlik T., Drochytek V., Hruda M. (2023). Twenty years of experience with less radical fertility-sparing surgery in early-stage cervical cancer: Pregnancy outcomes. Gynecol. Oncol..

[77] van der Plas R.C.J., Bos A.M.E., Jürgenliemk-Schulz I.M., Gerestein C.G., Zweemer R.P. (2021). Fertility-sparing surgery and fertility preservation in cervical cancer: The desire for parenthood, reproductive and obstetric outcomes. Gynecol. Oncol..

[78] Kuznicki M.L., Chambers L.M., Morton M., Son J., Horowitz M., Crean-Tate K.K., Hackett L., Rose P.G. (2021). Fertility-Sparing Surgery for Early-Stage Cervical Cancer: A Systematic Review of the Literature. J. Minim. Invasive Gynecol..

[79] Ronsini C., Solazzo M.C., Molitierno R., De Franciscis P., Pasanisi F., Cobellis L., Colacurci N. (2023). Fertility-Sparing Treatment for Early-Stage Cervical Cancer ≥ 2 cm: Can One Still Effectively Become a Mother? A Systematic Review of Fertility Outcomes. Ann. Surg. Oncol..

[80] Somigliana E., Mangili G., Martinelli F., Noli S., Filippi F., Bergamini A., Bocciolone L., Buonomo B., Peccatori F. (2020). Fertility preservation in women with cervical cancer. Crit. Rev. Oncol. Hematol..

[81] Sabeena S., Kuriakose S., Damodaran B., Ravishankar N., Arunkumar G. (2020). Human papillomavirus (HPV) DNA detection in uterine cervix cancer after radiation indicating recurrence: A systematic review and meta-analysis. J. Gynecol. Oncol..

[82] Adiga D., Eswaran S., Pandey D., Sharan K., Kabekkodu S.P. (2021). Molecular landscape of recurrent cervical cancer. Crit. Rev. Oncol. Hematol..

[83] Aisagbonhi O., Zare S.Y., Hasteh F., Binder P., Roma A.A., Fadare O. (2022). PTEN Loss and ARID1A Mutation in an HPV-positive Metastatic Adenocarcinoma Diagnosed Almost 18 yr After an Intact Cone Excision for Endocervical Adenocarcinoma In Situ. Int. J. Gynecol. Pathol..

[84] Chen Y., Zhu Y., Wu J. (2021). Prognosis of Early Stage Cervical Cancer according to Patterns of Recurrence. Cancer Manag. Res..

[85] Kim J.H., Park J.Y., Kim D.Y., Kim Y.M., Kim Y.T., Nam J.H. (2014). Long-term outcomes after fertility-sparing laparoscopic radical trachelectomy in young women with early-stage cervical cancer: An Asian Gynecologic Cancer Group (AGCG) study. J. Surg. Oncol..

[86] Plaikner A., Siegler K., Hertel H., Jacob A., Petzel A., Schubert M., Blohmer J.U., Böhmer G., Marnitz S., Ragosch V. (2023). Fertility sparing therapy in women with lymph node negative cervical cancer > 2cm—Oncologic and fertility outcomes of neoadjuvant chemotherapy followed by radical vaginal trachelectomy. Int. J. Gynecol. Cancer.

[87] Zusterzeel P.L., Pol F.J., van Ham M., Zweemer R.P., Bekkers R.L., Massuger L.F., Verheijen R.H. (2016). Vaginal Radical Trachelectomy for Early-Stage Cervical Cancer: Increased Recurrence Risk for Adenocarcinoma. Int. J. Gynecol. Cancer..

[88] Schuurman T., Zilver S., Samuels S., Schats W., Amant F., van Trommel N., Lok C. (2021). Fertility-Sparing Surgery in Gynecologic Cancer: A Systematic Review. Cancers.

[89] Tesfai F.M., Kroep J.R., Gaarenstroom K., De Kroon C., Van Loenhout R., Smit V., Trimbos B., Nout R.A., van Poelgeest M.I.E., Beltman J.J. (2020). Fertility-sparing surgery of cervical cancer > 2 cm (International Federation of Gynecology and Obstetrics 2009 stage IB1-IIA) after neoadjuvant chemotherapy. Int. J. Gynecol. Cancer.

[90] Li X., Jiang Z., Lu J., Chen X., Ge H., Wu X., Li J. (2023). Neoadjuvant chemotherapy followed by radical trachelectomy versus upfront abdominal radical trachelectomy for patients with FIGO 2018 stage IB2 cervical cancer. Gynecol. Oncol..

[91] Ronsini C., Solazzo M.C., Bizzarri N., Ambrosio D., La Verde M., Torella M., Carotenuto R.M., Cobellis L., Colacurci N., De Franciscis P. (2022). Fertility-Sparing Treatment for Early-Stage Cervical Cancer ≥ 2 cm: A Problem with a Thousand Nuances-A Systematic Review of Oncological Outcomes. Ann. Surg. Oncol..

[92] Viveros-Carreño D., Rodriguez J., Rendon Pereira G.J., Slama J., Halaska M.J., Robova H., Pareja R. (2022). Fertility-sparing surgery after neo-adjuvant chemotherapy in women with cervical cancer larger than 4 cm: A systematic review. Int. J. Gynecol. Cancer.

[93] Baiocchi G., Tsunoda A.T., Guitmann G., Vieira M.A., Zanvettor P.H., Silvestre J.B.C.H., Santos M.H., Sacramento R.M.M., de Araujo E.O., Lopes R.H. (2022). Brazilian Society of Surgical Oncology consensus on fertility-sparing surgery for cervical cancer. J. Surg. Oncol..

[94] Hruda M., Robova H., Rob L., Halaska M.J., Drozenova J., Pichlik T., Malikova H. (2021). Twenty years of experience with less radical fertility-sparing surgery in early-stage cervical cancer: Oncological outcomes. Gynecol. Oncol..

[95] Corrado G., Anchora L.P., Bruni S., Sperduti I., Certelli C., Chiofalo B., Giannini A., D’Oria O., Bizzarri N., Legge F. (2023). Patterns of recurrence in FIGO 2018 stage IB1-IB2 cervical cancer: Comparison between minimally invasive and abdominal radical hysterectomy. Eur. J. Surg. Oncol..

[96] Eriksen D.O., Jensen P.T., Schroll J.B., Hammer A. (2022). Human papillomavirus vaccination in women undergoing excisional treatment for cervical intraepithelial neoplasia and subsequent risk of recurrence: A systematic review and meta-analysis. Acta Obstet. Gynecol. Scand..

[97] Kechagias K.S., Kalliala I., Bowden S.J., Athanasiou A., Paraskevaidi M., Paraskevaidis E., Dillner J., Nieminen P., Strander B., Sasieni P. (2022). Role of human papillomavirus (HPV) vaccination on HPV infection and recurrence of HPV related disease after local surgical treatment: Systematic review and meta-analysis. BMJ.

[98] Lukic A., Rossi S., Frega A., Ruscito I., Bianchi P., Nobili F., Caserta D., Vecchione A. (2021). Prognostic role of immunohistochemical overexpression of the p16 protein in women under the age of 35 and diagnosed with HSIL (CIN2) subjected to “cervix sparing” excision. Eur. Rev. Med. Pharmacol. Sci..

[99] Jeannot E., Latouche A., Bonneau C., Calméjane M.A., Beaufort C., Ruigrok-Ritstier K., Bataillon G., Larbi Chérif L., Dupain C., Lecerf C. (2021). Circulating HPV DNA as a Marker for Early Detection of Relapse in Patients with Cervical Cancer. Clin. Cancer Res..

[100] Harima Y., Sawada S., Nagata K., Sougawa M., Ohnishi T. (2002). Human papilloma virus (HPV) DNA associated with prognosis of cervical cancer after radiotherapy. Int. J. Radiat. Oncol. Biol. Phys..

[101] Song Y.J., Kim J.Y., Lee S.K., Lim H.S., Lim M.C., Seo S.S., Kang S., Lee D.O., Park S.Y. (2011). Persistent human papillomavirus DNA is associated with local recurrence after radiotherapy of uterine cervical cancer. Int. J. Cancer.

[102] Noventa M., Ancona E., Cosmi E., Saccardi C., Litta P., D’Antona D., Nardelli G.B., Gizzo S. (2014). Usefulness, methods and rationale of lymph nodes HPV-DNA investigation in estimating risk of early stage cervical cancer recurrence: A systematic literature review. Clin. Exp. Metastasis.

[103] Vintermyr O.K., Iversen O., Thoresen S., Quint W., Molijn A., de Souza S., Rosillon D., Holl K. (2014). Recurrent high-grade cervical lesion after primary conization is associated with persistent human papillomavirus infection in Norway. Gynecol. Oncol..

[104] Bai H., Liu J., Wang Q., Feng Y., Lou T., Wang S., Wang Y., Jin M., Zhang Z. (2018). Oncological and reproductive outcomes of adenocarcinoma in situ of the cervix managed with the loop electrosurgical excision procedure. BMC Cancer.

[105] Byun J.M., Jeong D.H., Kim Y.N., Jung E.J., Lee K.B., Sung M.S., Kim K.T. (2018). Persistent HPV-16 infection leads to recurrence of high-grade cervical intraepithelial neoplasia. Medicine.

[106] Bogani G., Pinelli C., Chiappa V., Martinelli F., Lopez S., Ditto A., Raspagliesi F. (2020). Age-specific predictors of cervical dysplasia recurrence after primary conization: Analysis of 3,212 women. J. Gynecol. Oncol..

[107] Bogani G., DI Donato V., Sopracordevole F., Ciavattini A., Ghelardi A., Lopez S., Simoncini T., Plotti F., Casarin J., Serati M. (2020). Recurrence rate after loop electrosurgical excision procedure (LEEP) and laser Conization: A 5-year follow-up study. Gynecol. Oncol..

[108] Spinillo A., Dominoni M., Boschi A.C., Sosso C., Fiandrino G., Cesari S., Gardella B. (2020). Clinical Significance of the Interaction between Human Papillomavirus (HPV) Type 16 and Other High-Risk Human Papillomaviruses in Women with Cervical Intraepithelial Neoplasia (CIN) and Invasive Cervical Cancer. J. Oncol..

[109] Spinillo A., Dominoni M., Boschi A.C., Cesari S., Fiandrino G., Gardella B. (2020). The relationship of human papillomavirus infection with endocervical glandular involvement on cone specimens in women with cervical intraepithelial neoplasia. Gynecol. Oncol..

[110] Kulkarni A., Covens A., Durand N., Ghorab Z., Gien L.T., Osborne R., Vicus D., Kupets R. (2023). Role of HPV in the Prediction of Persistence/Recurrence After Treatment for Cervical Precancer. J. Obstet. Gynaecol. Can..

[111] Zang L., Hu Y. (2021). Risk factors associated with HPV persistence after conization in high-grade squamous intraepithelial lesion. Arch. Gynecol. Obstet..

[112] Han L., Zhang B. (2023). Can prophylactic HPV vaccination reduce the recurrence of cervical lesions after surgery? Review and prospect. Infect. Agent. Cancer.

[113] Jentschke M., Kampers J., Becker J., Sibbertsen P., Hillemanns P. (2020). Prophylactic HPV vaccination after conization: A systematic review and meta-analysis. Vaccine.

